# Screening of Emerging Pollutants (EPs) in Estuarine Water and Phytoremediation Capacity of *Tripolium pannonicum* under Controlled Conditions

**DOI:** 10.3390/ijerph18030943

**Published:** 2021-01-22

**Authors:** Ariel E. Turcios, Marie Hielscher, Bernardo Duarte, Vanessa F. Fonseca, Isabel Caçador, Jutta Papenbrock

**Affiliations:** 1 Institute of Botany, Leibniz University Hannover, Herrenhäuserstr. 2, D-30419 Hannover, Germany; mar.hielscher@web.de (M.H.); papenbrock@botanik.uni-hannover.de (J.P.); 2 MARE-Marine and Environmental Sciences Centre, Faculdade de Ciências da Universidade de Lisboa, Campo Grande, 1749-016 Lisbon, Portugal; baduarte@fc.ul.pt (B.D.); vffonseca@fc.ul.pt (V.F.F.); micacador@fc.ul.pt (I.C.); 3 Departamento de Biologia Vegetal, Faculdade de Ciências da Universidade de Lisboa, Campo Grande, 1749-016 Lisbon, Portugal; 4 Departamento de Biologia Animal, Faculdade de Ciências da Universidade de Lisboa, Campo Grande, 1749-016 Lisbon, Portugal

**Keywords:** emerging pollutants, halophytes, organic pollution, phytoremediation, xenobiotics

## Abstract

The increasing number of pharmaceuticals in the environment and their difficult biodegradation, can lead to bioaccumulation in different trophic compartments. Their bioaccumulation can have negative consequences, especially in the generation of bacterial resistance by antibiotics, but also in the impairment of plant and animal metabolism. The Tejo estuary in Portugal is the habitat for many plant and animal species, which are also prone to this type of contamination. Therefore, in the present study different classes of emerging pollutants (EPs) were surveyed in water samples in the Tejo estuary, including antibiotics, anticonvulsants, antidepressants, lipid-lowering drugs, anti-inflammatory drugs, beta-blockers and analgesics. According to the results, only four compounds were detected in water samples collected at the three selected salt marshes, including carbamazepine, fluoxetine hydrochloride, venlafaxine hydrochloride and acetaminophen. Having the detected substances as a basis, a subsequent study was performed aiming to investigate the uptake and biodegradation capacity of halophytes, using *Tripolium pannonicum* as a model plant cultivated under controlled conditions with different concentrations of the found EPs. This experimental approach showed that *T. pannonicum* was able to uptake and degrade xenobiotics. Moreover, the application of sulfamethazine, as a model antibiotic, showed also that this species can uptake and degrade this compound, although the degradation rate and process proved to be compound-specific. This was also confirmed using crude plant extracts spiked with the different EPs. Thus this species is a potential candidate for the remediation of marine water and sediments contaminated with environmentally-significant EPs.

## 1. Introduction

In recent years, environmental pollution concerns have increased due to the presence of different and emerging contaminants in the environment that pose significant threats to the environment and human health. A large number of organic pollutants have been found worldwide in surface waters, estuaries, groundwater, and soils, among other matrices [1,2,3]. One of the main reasons for the increase of chemical contaminants in the environment is the high demand for these products and their misuse. For example, the production of chemicals hazardous to health in the EU was 222.6 million tons in 2018 [4]. Related to this, there has also been an increase in micro-pollutants or emerging pollutants (EPs). Emerging pollutants are a vast and expanding number of compounds of anthropogenic origin that are commonly present in the environment. These contaminants include pharmaceuticals, pesticides, steroid hormones, personal care products, industrial and household products, metal nanoparticles, surfactants, industrial additives, paints, flame retardants and solvents. Many of these compounds are used and released into the environment, leading to acute and chronic toxicity events, metabolic and endocrine disruption in humans and aquatic wildlife [5,6,7], and the development of bacterial pathogen resistance, impairment of marine primary productivity [8,9,10,11], among other effects [12]. Mainly due to microbial resistance, antibiotics found in different sources have attracted the attention of the scientific community. Worldwide, drug consumption surveys between 2002 and 2009 show a significant increase in consumption of the compounds [13]. According to Klein et al. [14], antibiotic consumption worldwide increased by 65% between 2000 and 2015. This increase inevitably leads to an increase in concentration in the environment and thus a higher probability of antibiotic resistance of organisms as well as direct impacts in the organism’s physiology. 

The combination of the increasing amount of pharmaceuticals in the environment and their low biodegradability, high persistence in the environment and passive particle behaviour within estuarine systems can lead in the long term to their bioaccumulation [15]. The bioaccumulation of EPs in plants or animals can be due to chronic exposure of various pharmaceutical substances, in particular from the lowest to the upper trophic levels and ultimately human beings. The above observations on the impact of exposure to xenobiotics (XBs) on environmental processes may, over time, have far-reaching consequences, given the close integration of biological processes. Various organisms living in a XB contaminated environment can uptake these compounds and thus can function as natural biofilters [16,17,18]. Since some plants and animals that live in contaminated water are used for human consumption, long-term health effects on human health may occur. For this reason, it is relevant to investigate the contaminants dynamics in the abiotic and biotic environmental compartments as well as the ability of some plants to uptake, modify and biodegrade these contaminants. In this sense, plants can be used as a biofilter to remediate waters from undesirable pollutants. This process, also known as phytoremediation, is considered to be inexpensive and environmentally friendly compared to chemical or physical treatment processes and therefore represents a highly forward-looking field of research [19,20]. 

The main objective of this study is to identify EPs and determine the uptake and phytoremediation capacity of halophyte plants. The classes of drugs relevant in this study include antibiotics, anticonvulsants, antidepressants, lipid-lowering drugs, non-steroidal anti-inflammatory drugs, beta-blockers and analgesics. The largest proportion of the substances investigated here belongs to the antibiotics. In order to perform an environmentally relevant survey, water samples were collected in the vicinity of three different salt marshes known to have different contamination degrees [21]. Samples were screened for XBs by liquid chromatography coupled to mass spectrometry (LC-MS). Having the obtained values in mind, and in order to study the uptake and degradation capacity of XBs by plants, the halophyte *Tripolium pannonicum* was cultivated under controlled conditions containing four XBs detected in the water samples: carbamazepine (CBZ), fluoxetine hydrochloride (FLU), venlafaxine hydrochloride (VEN) and acetaminophen (APAP). Sulfamethazine (SMT) was also tested as a model antibiotic. 

## 2. Materials and Methods 

### 2.1. Sample Collection and Study Site Description 

**Figure 1:**
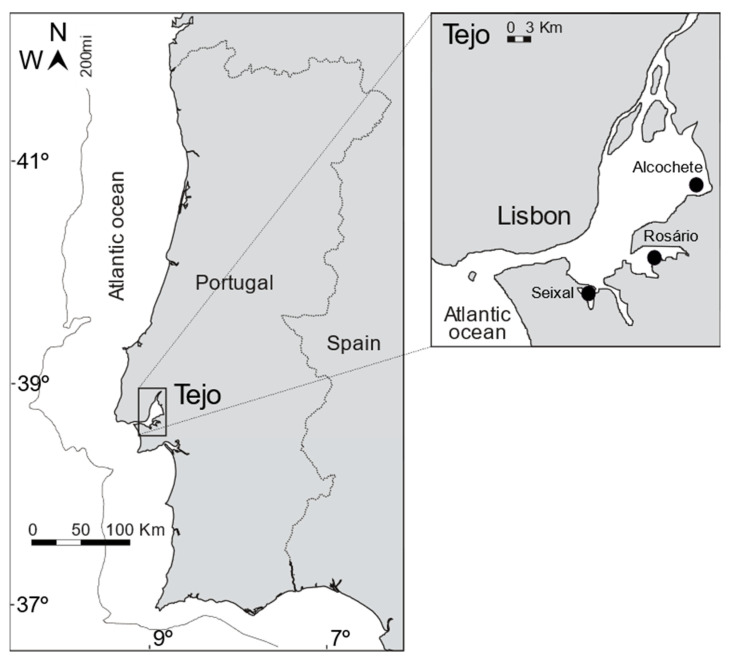
Overview of the sampling areas in the Tejo estuary. Black circles indicate the three sampling sites, namely Alcochete, Rosário and Seixal.

Sampling occurred at the end of the growing season (summer 2019) in three salt marshes of the Tejo Estuary (Figure 1). The Hortas salt marsh (Alcochete; 38°45.661′ N, 8°56.116′ W) is located in the middle estuary, adjacent to the Tagus Estuary Natural Reserve, being considered a reference site with very low anthropogenic pressure levels [21]. The Rosário salt marsh (38°40.161′ N, 9°00.198′ W) and the Seixal Bay salt marsh (38°38.313′ N, 9°07.191′ W) are located in the lower estuary, in the vicinity of highly urbanized and industrialized areas being subjected to high anthropogenic pressure levels [22,23]. All three salt marshes are hydrodynamically similar, being subjected to tidal flooding twice per day, and dominated by the halophyte species *Spartina maritima* in the lower marsh (circa 12% coverage), *Halimione portulacoides* in the mid-upper marsh (circa 35% coverage), and *Sarcocornia fruticosa* in the upper marsh (circa 20% coverage) [24]. All salt marshes have recently been invaded by a non-indigenous plant species (*Spartina patens*) with already a high colonization area [21]. Five surface water samples (first 50 cm depth) were collected during high tide in each of the above mentioned salt marshes, using 1.5 L high-density polyethylene containers, acidified to pH 2 with p.a. formic acid (CH_2_O_2_), transported on ice and in the dark to the laboratory, and preserved frozen (−20 °C). All used plastic ware was pre-washed in 10% p.a. nitric acid (HNO_3_) and triple rinsed in ultrapure water.

### 2.2. Water Sample Processing 

Water samples collected for pharmaceutical quantification were extracted, purified, and concentrated following a protocol adapted from Pereira et al. [25] and Sousa et al. [26]. Briefly, samples (1 L) were filtered through three filters (Whatman GF/C™, and polyamide membrane filters of 0.45 μm and 0.2 μm), extracted with OASIS HLB cartridges, washed with methanol:water (10:90, 5 mL) and dried for 15 min at low vacuum pressure. The elution of the compounds was then accomplished with methanol (6 mL) and the extract dried under a nitrogen atmosphere, at 40 °C. 

### 2.3. Plant Cultivation under Controlled Conditions 

The seeds of *Tripolium pannonicum* (Jacq.) Dobrocz. were collected at the North Sea, Germany (53°29′13″ N; 8°03′16″ E). The agronomic handling from sowing through transplanting was carried out as described by Buhmann et al. [27]. From June 2019 to July 2019, *T. pannonicum* plants were grown under different concentrations (2 and 5 mg L^−1^ XB) of the four XBs found at the different sampling sites (APAP: analgesic and antipyretic, CBZ: anticonvulsant, FLU: antidepressant, VEN: antidepressant) and SMT as a model antibiotic. SMT was used as model antibiotic because it is very stable under different environmental conditions and it does not adhere easily to surfaces, and it is also highly used in livestock. In addition to the abovementioned treatments (2 and 5 mg L^−1^ XB), another treatment was used as control (three biological replicates) where *T. pannonicum* was cultivated but without the addition of XBs. This was carried out with individual XBs as well as using a mixture of all five XBs to study interaction effects. All plants were grown for five weeks after transplanting in hydroponic conditions. These experiments were conducted in a greenhouse at the Institute of Botany, Leibniz University Hannover, Germany (52°23′42″ N; 9°42′13″ E), with temperatures between 14 °C (minimum temperature during the night) and 35 °C (maximum temperature during the day). Plants were exposed to 12 h of artificial light (sodium vapour lamps, SON-T Agro 400, Philips). Light intensity ranged from 65 µmol·m^−2^·s^−1^ to 850 µmol·m^−2^·s^−1^ depending on the time of the day and the weather conditions. Each polypropylene container had 2 L solution containing 606 mg·L^−1^ KNO_3_, 944 mg·L^−1^ Ca(NO_3_)_2_·4H_2_O, 230 mg·L^−1^ NH_4_H_2_PO_4_, 246 mg·L^−1^ MgSO_4_·7H_2_O, 3.73 mg·L^−1^ KCl, 1.55 mg·L^−1^ H_3_BO_3_, 0.34 mg·L^−1^ MnSO_4_·H_2_O, 0.58 mg·L^−1^ ZnSO_4_·7H_2_O, 0.12 mg·L^−1^ CuSO_4_·5H_2_O, 0.12 mg·L^−1^ MoNa_2_O_4_·2H_2_O, and 9.16 mg·L^−1^ Fe-EDDHA (0.56 mg·L^−1^ Fe). The water was constantly aerated by small compressors and one air stone in the middle of each tank (Eheim, Deizisau, Germany). The hypocotyl was fixed with soft foam in 35 mm holes. The water level was adjusted constantly in each container to compensate for evapotranspiration. Each experimental unit consisted of three plants per container, with three replicates (containers) per treatment. To study the degradation of XBs due to environmental conditions, controls with a concentration of 5 mg L^−1^ each were used but without plants. After harvesting, the aerial and root fresh biomass was determined separately using an Acculab balance (ATL-623-I, Acculab, Sartorius Group, Göttingen, Germany), and then the different parts of the plants (shoots and roots) were washed with deionized water, water was removed using blotting paper and immediately frozen in liquid nitrogen. Plant samples were stored at −80 °C before extraction.

### 2.4. Xenobiotic Degradation Using Crude Plant Extracts

In order to study the potential of the halophyte *T. pannonicum* in degrading XBs, an enzymatic test was done. In this study, the enzymatic degradation of the XBs: SMT, APAP, CBZ, VEN and FLU was addressed using plant extract of the halophyte *T. pannonicum* as a model plant. The plants were cultivated under hydroponic conditions as mentioned above but without XBs in the culture medium. Plants were harvested and stored at −80 °C before the enzyme extraction. The extraction was carried out according to Turcios and Papenbrock [28]. Briefly, 3 g of frozen plant material (−80 °C) was weighed and extracted with 15 mL TRIS/HCl buffer (20 mM, pH 7). The samples were vortexed for 10 min and then centrifuged for 15 min at 15,700× *g* (5415 R centrifuge, Eppendorf, Hamburg, Germany) at 4 °C. The supernatant was then transferred to a new reaction vessel. In each case, three technical replicates were used. Then, the different XBs were first diluted and added to the plant extract so that each treatment ended up with a volume of 500 μL and a XB concentration of 5 mg L^−1^. In the first test, individual XBs were added to the plant extract. In a second experiment, a mixture of all five substances was prepared and added to the plant extract. Additionally, a variation with denatured plant extract (15 min, 95 °C) was prepared for each treatment to test whether the degradation of XBs depends on the enzyme activity. A total of 42 samples (three replicates per treatment) with a volume of 500 μL each were made. Subsequently, all samples were incubated at 37 °C for 7 d. After incubation, all samples were first vortexed and then diluted with methanol LC-MS grade from a starting concentration of 5 mg L^−1^ to the desired concentration of 250 ng mL^−1^. Subsequently, the respective XB concentrations of the samples were determined using liquid chromatography coupled to a mass spectrometer LC-MS (see below).

### 2.5. Extraction of Xenobiotics from Plant Samples 

The extraction was performed according to Turcios et al. [18]. Briefly, the samples stored at −80 °C were crushed and 100 mg of fresh sample was weighted in 2 mL reaction tubes. For the extraction, acid hydrolysis was performed. Extraction buffer was added (1000 µL, 1% formic acid in MeOH *v*/*v*). The samples were shaken at 1000 min^−1^ at room temperature for 4 h (Eppendorf^®^ Thermomixer Compact, Hamburg, Germany). Then 1000 µL of hydrolysis buffer was added (10% HCl in MeOH *v*/*v*) and heated at 85 °C for 40 min. After cooling, the samples were centrifuged for 5 min at 10,000 min^−1^ (9279× *g*). 500 µL of the supernatant was diluted in 500 µL 80% MeOH. Internal standard (simeton) was added to the samples to reach a final concentration of 100 ng mL^−1^.

### 2.6. LC/MS Conditions, Quantification and Linearity 

Samples were analyzed by a high-performance liquid chromatography (HPLC) system (Shimadzu, Canby, USA), equipped with two solvent delivery units (LC-20AD_XR_), an autosampler (SIL-20AC_XR_), a photodiode array detector (SPD-M20A), and a column oven (CTO-20AC). Analytes were separated using a Vertex Plus column (250 × 4 mm, 5 µm pore size, packing material ProntoSIL 120-5 C18-H) with a corresponding precolumn (Knauer, Berlin, Germany). The analytes were separated and identified within 32 min, therefore a total run time of 40 min per sample was allowed. A binary gradient with a flow rate of 0.80 mL·min^−1^ was used. Mobile phase A contained 2 mM ammonium acetate in ultrapure water. Mobile phase B contained 2 mM ammonium acetate in MeOH. For separation, the initial mobile phase containing 10% B was increased linearly to 90% B in 35 min, which was held for 2 min before decreasing linearly to 10% B in 1 min and holding for an additional 2 min to allow re-equilibration of the column. The eluted compounds were analyzed by UV/Vis spectra from 190–800 nm using a deuterium lamp. The HPLC system was coupled to an AB Sciex Triple TOF 4600 mass spectrometer (AB Sciex Deutschland GmbH, Darmstadt, Germany). Positive electrospray ionization (ESI) was used at a nebulizer temperature of 600 °C and an ion spray voltage floating of 4500 V. Mass spectra in the range of 120–930 Da were measured in the TOF range, in addition, MS/MS spectra from 50–800 Da at a collision energy of 30 V were recorded.

Quantitation was based on a detector response defined as the area ratio of the base peak ion to the base peak ion of the internal standard (MultiQuant™ Software, AB Sciex). The calibration curve was constructed by plotting the area ratio of the ion response of the external standard (17 different standards) and the internal standard (simeton) against the ratio of the spiked concentrations. The internal standard simeton was used to compensate for the variation of the volumes of the final extracts and to check the instrumental performance. The limits of detection (LOD) and quantification (LOQ) were determined according to the DIN 32645 (Table S1). For identification and quantitation of non-target metabolites, MasterView™ Software (AB Sciex) was used. Linearity was tested with concentrations from 5 to 200 ng·mL^−1^ external standards containing a concentration of 100 ng·mL^−1^ of simeton as an internal standard. 

### 2.7. Statistical Analysis 

All statistical analyses were conducted using R, version 3.1.1 (R Core Team, Vienna, Austria) and InfoStat software, version 2016e (InfoStat Team, Cordoba, Argentina). The effects of the two main factors: XBs and concentrations, on the different parameters were analyzed through a two-way analysis of variance (ANOVA). Xenobiotic degradation by crude plant extract was analyzed by one-way ANOVA. The Tukey multiple comparison test with a significance level of = 0.05 was done to determine which means differ from the rest. The control (plants grown in the absence of XBs) was not considered within the statistical analysis, where no trace of XBs was found. The control was used only as a reference for biomass production.

## 3. Results and Discussion 

### 3.1. Standards Mass Spectra Analysis

**Figure 2:**
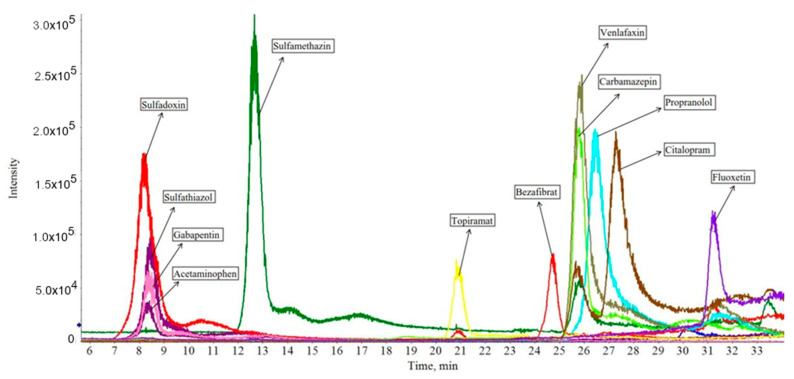
Chromatogram of XB standards. Overview of retention times and intensities of the identified standards based on the Extracted Ion Chromatogram.

In order to identify and quantify the greatest number of XBs in the samples, different standards were analyzed. From a total of 17 examined standards, 14 could be clearly identified with our method and equipment. The detection of the following substances was possible at low concentrations (below 100 ng mL^−1^): azithromycin dihydrate, bezafibrate, carbamazepine, citalopram hydrobromide, fluoxetine hydrochloride, gabapentin, propranolol hydrochloride, sulfamethazine and venlafaxine hydrochloride. Acetaminophen and gemfibrozil could only be clearly identified at higher concentrations (500 ng mL^−1^). Tetracycline, oxytetracycline and ibuprofen could not be detected even at higher concentrations (500–1000 ng mL^−1^). Figure 2 shows the chromatogram of a standard mixture (in MeOH, LC-MS grade) in which each standard was applied at a concentration of 100 ng mL^−1^. 

Although each standard had the same concentration, the intensities differ significantly from each other due to the intrinsic properties of each substance (Table 1). Azithromycin dihydrate is not included in Figure 2 because of its difficult detectability.

### 3.2. Xenobiotic Concentration in Estuarine Water Samples

The analysis of estuarine water samples revealed the presence of CBZ, FLU, VEN and APAP. Except for FLU, the content of XBs in the contaminated area (Seixal) is highest, decreasing in the moderately contaminated area (Rosário) and are no longer detectable in the pristine area (Alcochete). The level of FLU decreases only slightly in the low contaminated and uncontaminated area (Table 2). 

### 3.3. Exposure of the Halophyte Tripolium Pannonicum to Xenobiotics 

With the aim of studying the capacity of *T. pannonicum* to uptake and degrade the different XBs, as well as the effect of these XBs in the biomass yield, following identification of XBs in one of the largest estuaries in Europe, the Tejo estuary, *T. pannonicum* was cultivated under controlled conditions with different XB concentrations in the culture medium. This halophyte was chosen as a model plant because it is a species with a broad distribution, found naturally on many estuaries including the Tejo estuary, and also because it is a plant species that has a high potential to be used in the phytoremediation process [28].

#### 3.3.1. Influence of Xenobiotics on Biomass Yield

**Figure 3:**
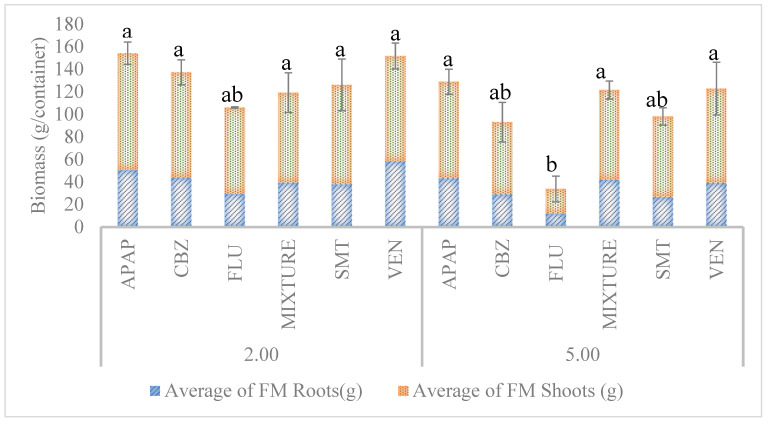
Influence of xenobiotics on the total fresh mass in *Tripolium pannonicum* (FM = fresh mass; APAP = acetaminophen; CBZ = carbamazepine; FLU = fluoxetine; MIXTURE = mixture of all five xenobiotics; SMT = sulfamethazine; VEN = venlafaxine; 2 = 2 mg L^−1^; 5 = 5 mg L^−1^. Columns show average ± standard error of three biological replicates per treatment. Values with the same letter are not significantly different (*p* > 0.05)).

According to the analysis of variance, it could be shown that the biomass yield was significantly dependent on the XB treatment as well as on the XB concentration (Figure 3). 

The plants treated with 2 mg L^−1^ APAP showed the highest biomass yield (154.23 ± 16.99 g/container). On the other hand, the plants treated with 5 mg L^−1^ FLU presented the lowest total biomass (33.77 ± 16.99 g/container). The control treatment without the addition of XBs resulted in a total biomass of 104.88 ± 7.78 g/container. Based on this, all the XB treatments with a concentration of 2 mg L^−1^ produced higher biomass compared with the control but at 5 mg L^−1^ the biomass yield decreased significantly, namely for CBZ, FLU and SMT. This higher biomass yield at relatively low XB concentrations (2 mg L^−1^) could be due to developed biochemical mechanisms to deal with the stress, such as alteration in the cell wall, cytoskeleton and membrane structure [18], while at higher concentrations the decrease in biomass production could be, as reported by Turcios et al. [29], part of a higher stress response in plants caused by some antibiotics, which depends on the antimicrobial itself, concentration and plant species. In line with this, Turcios et al. [28] also reported a biomass decrease in *Chenopodium quinoa* using the antibiotic SMT. In the case of SMT, it interferes with folic acid synthesis via inhibition of the enzymatic conversion of pteridine and p-aminobenzoic acid (PABA) to dihydropteroic acid by competing with PABA for binding to dihydrofolate synthetase, an intermediate of tetrahydrofolic acid synthesis, required for purines and deoxythymidine monophosphate synthesis [30,31]. This can decrease the amount of folate in the plant and, consequently, may lead to a decrease in biomass yield. 

As stated above, the lowest biomass productions were obtained using FLU. Some studies suggest that FLU is a potent serotonin uptake inhibitor, by blocking the reuptake transporter protein [32]. Serotonin has been found in a wide range of plant species. Similar to the multiple roles played by serotonin in animal cells, in plants this molecule has also been implicated in an array of physiological functions that are purportedly related to growth regulation, flowering, xylem sap exudation, ion permeability, and plant morphogenesis [33]. The presence of this antidepressant in plants can therefore have implications in the aquatic life. This was previously reported for the marine diatom *Phaeodactylum tricornutum* [9], showing that this compound severy impaired the photochemical metabolism and biomass production of this autotrophic specie. For example, Ford et al. [34] reported that they found significant effects on foot detachment in snail species and righting times at the high (1 mg L^−1^) concentration of FLU, however, this concentration is higher than might be expected from wastewater treatment plants. The concentrations of antidepressants detected in the aquatic environment can vary based on drug, country and region whereby they are prescribed [34]. Moreover, some plant species are tolerant to XBs either by selective uptake mechanisms, dissipation of the xenobiotic in the rhizosphere or by a release of stress response hormones [35]. 

#### 3.3.2. Uptake of Xenobiotics by Tripolium Pannonicum and Their Distribution in the Plant

In all treatments, the different XBs could be detected in the plant material. Accordingly, the plant species *T. pannonicum* can uptake all the tested substances through the roots and translocate them to the shoots (Table 3). According to the ANOVA test, the concentration in the plant tissues is significantly dependent on the XB and the initial concentration in the culture medium. In the shoot samples, the highest amount of xenobiotic (13.57 ± 0.71 µg g^−1^ FM) could be detected for the plants exposed to CBZ treatment at an initial concentration of 5 mg L^−1^ in the culture medium. In the other initial concentrations, CBZ remained in higher concentrations compared to the rest of the XBs. All other XBs showed significantly lower concentrations of the parent substance, which did not differ significantly from each other. The lowest concentration (0.02 ± 0.00 µg g^−1^ FM) was found in the FLU treatment with an initial concentration of 0.40 mg L^−1^ in the culture medium.

Comparing the amount of XBs that could be quantified in the roots, it can be observed that the plants exposed to SMT 5 mg L^−1^ showed the highest SMT root concentration (68.96 ± 3.28 µg g^−1^ FM), while the lowest concentration was detected in the plants exposed to FLU. It should also be noted that the higher the initial concentration in the culture medium, the higher the amount of XB quantified in the roots (Table 4). Therefore, these XBs could be taken up by plants in a passive way, subsequently, the translocation within the plant can occur by mass flow and diffusion [36].

**Figure 4:**
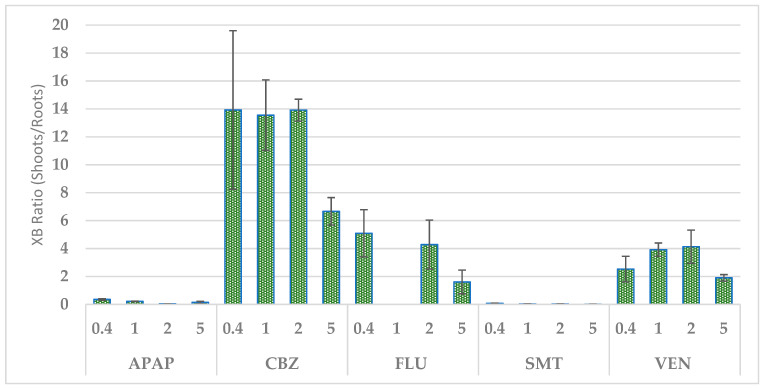
Xenobiotic concentration ratio in the plant biomass. APAP = acetaminophen; CBZ = carbamazepine; FLU = fluoxetine; SMT = sulfamethazine; VEN = venlafaxine. The values 5 and 2 on the X axis represent the singly xenobiotic concentration in the culture medium in mg L^−1^, and the values 1 and 0.4 represent each xenobiotic concentration in a mixture of the 5 xenobiotics in mg L^−1^. Columns show average ± standard error of three biological replicates per treatment.

In the APAP and SMT treatments, the measured root concentrations were higher than in the shoot samples. The xenobiotics CBZ, FLU and venlafaxine (VEN) showed an opposite distribution pattern, with higher concentrations observed in the shoot samples than in the root samples, indicating a higher translocation (Figure 4).

#### 3.3.3. Mass Balance

**Figure 5:**
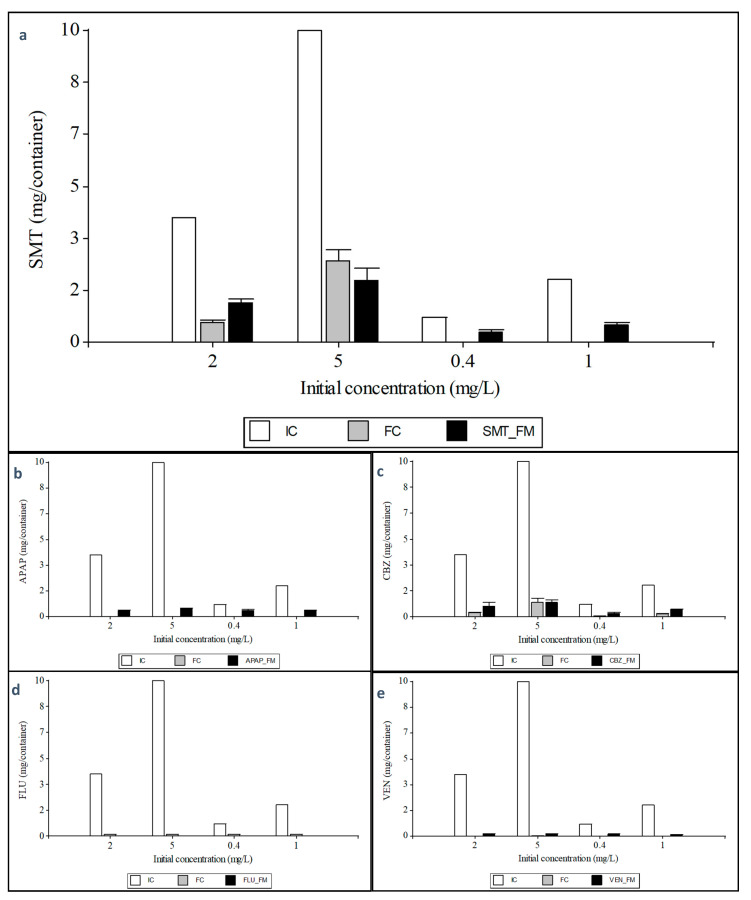
Mass balance of xenobiotics in the culture system. SMT = Sulfamethazine (**a**); APAP = Acetaminophen (**b**); CBZ = Carbamazepine (**c**); FLU = Fluoxetine (**d**); VEN = Venlafaxine (**e**); IC = Initial content in the culture medium; FC = Final content in the culture medium; XB_FM = Xenobiotic content in the total fresh plant material. The values 2 and 5 on the *x*-axis represent the singly xenobiotic concentration and the values 0.4 and 1 represent each xenobiotic concentration in a mixture of the five xenobiotics in milligram per liter. Values show the mean ± standard error of three biological replicates per treatment.

This mass balance aims to understand how XBs are taken up by plants and degraded which is an application of conservation of mass in the system. For this purpose, the initial and final amount of XBs in the system, and the total amount in the plant material were considered. The quantity not found could have been degraded by plants, microorganisms or other environmental factors as discussed below. Regarding the total XB mass uptake by plants, significant differences could be detected among treatments, not only on the xenobiotic and but also on the XB initial exogenous concentration applied. The highest XB amount found in the plant material was observed for SMT with a value of 1.98 ± 0.12 mg/container when subjected to an initial concentration of 5 mg L^−1^ in the culture medium. FLU was found at very low concentrations in the plant tissues (Figure 5), and thus significantly different from the other treatments. 

The SMT treatment with an initial concentration of 10 mg/container (5 mg L^−1^), a decrease of 7.41 ± 0.18 mg/container was found in the culture medium during the plant cultivation, while 1.98 ± 0.21 mg was found in the whole plant tissues, which is approximately 26.42 ± 6.30% of the decreased amount. At an initial concentration of 4 mg/container (2 mg L^−1^), a decrease of 3.36 ± 0.18 mg/container was measured. In this treatment, 1.25 ± 0.21 mg/container was found in the plant tissues, which represents 37.38 ± 6.30%. The xenobiotic mixture treatment with an initial concentration of 2 mg/container SMT (1 mg L^−1^) showed a decrease of 2.00 ± 0.18 mg/container, while in the plant tissues an amount of 0.57 ± 0.21 mg/container was measured. This represents only 28.61 ± 6.30% of the total decrease in the solution, meaning that 71% of the parent compound was degraded. In the plants exposed to the mixture treatment and at a starting concentration of 0.80 mg/container SMT (0.4 mg L^−1^), a decrease of 0.80 ± 0.18 mg/container was found. The 0.30 ± 0.21 mg/container found in the plant accounts for 37.95 ± 6.30% of the total decrease. According to these results, it can be observed that SMT decreased about 100% in the treatments of a xenobiotic mixture where SMT concentrations were lower, although only a part of this was quantified in the plant material. The amount not quantified in this balance could have been degraded in the rhizosphere by different microorganisms, but it is most likely that most of it has been degraded by different enzymes in the plant. In line with this, Turcios et al. [18] also reported that only about 39% of the decreased total amount in the growth media was found in the plant tissues, meaning that about 61% could be degraded by plants. Each plant species has its mechanism to degrade organic contaminants [28], this includes selective uptake mechanisms, dissipation in the rhizosphere or release of stress response hormones. According to Mathews and Reinhold [35], there are three phases of plant metabolism of organic contaminants: Phase I, transformation of organic contaminants; Phase II, conjugation of parent contaminants and Phase I metabolites; and Phase III, sequestration or compartmentalization of Phase II metabolites. Burken [37] reported that hydroxylation is the most prevalent metabolic process for organic compounds in plants. N-glycosidation is also a likely mechanism of plant metabolism of sulfonamides [35]. Therefore, it is highly probable that the amount not found has been degraded by the plants and to confirm this a study with crude extract of *T. pannonicum* was also carried out, which is described later.

APAP treatment with an initial concentration of 10 mg/container (5 mg L^−1^) showed a decrease in the nutrient solution of 10.00 ± 0.00 mg/container. From this amount, 0.52 ± 0.07 mg/container could be quantified in the plant. The treatment with an initial concentration of 4 mg/container (2 mg L^−1^) resulted in a decrease of 4.00 ± 0.00 mg/container. Of this, 0.41 ± 0.07 mg/container could be detected in the plant, which represents only 10.23 ± 6.85% of the total decrease. When the plants were treated with the xenobiotic mixture, with an APAP concentration of 2 mg/container and 0.8 mg/container, APAP could not be detected in the culture medium at the end of the cultivation period, and only 0.38 ± 0.07 mg/container could be quantified in both treatments (Figure 5b). In this case, it can be confirmed that the degradation of this compound was not only due to the plants but possibly to other external factors. This could be affirmed since in the control containers (not containing plants), a decrease of the parent compound was also determined (data not shown). This suggests that the parent compound can transform and degrade partially due to external factors, including photolytic removal [38]. Moreover, microorganisms can play an important role in the rhizosphere which have established effective strategies involving specialized enzyme systems and metabolic pathways to access paracetamol as a carbon and energy source, producing some intermediate metabolites such as aromatic derivatives or organic acids [39,40].

The CBZ treatment showed a decrease in the parental substance of 9.09 ± 0.13 mg/container at an initial concentration of 10 mg/container. Of this amount, 0.92 ± 0.14 mg/container could be detected in the plant tissues, which represents 9.99 ± 6.62% of the total decrease. The treatment with 4 mg/container, resulted in a CBZ decrease of 0.69 ± 0.14 mg/container, which explains 18.26 ± 6.62% of the total decrease. The plants treated with the mixture of XBs, with 2 mg/container CBZ resulted in a decrease of 1.86 ± 0.13 mg/container. In the plant tissue, 0.42 ± 0.14 mg/container could be quantified, while the treatment with 0.80 mg/container CBZ resulted in a decrease of 0.75 ± 0.13 mg/container, where an amount of 0.18 mg/container was detected in the plant tissues (Figure 5c). In line with this, previous studies have been able to demonstrate the degradation of CBZ. For example, in studies on tomato plants, which were also cultivated under hydroponic conditions and a CBZ concentration of 0.5 mg L^−1^, it was shown that at least 33% of taken up CBZ was degraded. Besides, the experiment identified a total of 21 transformation products [41]. In other previous experiment carried out by Goldstein et al. [42], in which cucumber plants were exposed under hydroponic conditions to a CBZ concentration of 0.43 mg L^−1^, a CBZ degradation of more than 10% could be detected in the first 96 h. The experiment also showed that different drugs can influence each other in their uptake rate and metabolism.

Regarding VEN and FLU the amounts detected after the cultivation period were very low. For VEN, a decrease of 9.97 ± 0.00 mg/container was measured with an initial concentration of 10 mg/container. Of this, an amount of 0.12 ± 0.02 mg/container was quantified in the plant tissues. At a concentration of 4 mg/container (2 mg L^−1^), a decrease of 3.99 ± 0.00 mg/container was found, while in the plant tissues only 0.15 ± 0.02 mg/container could be measured. The mixture treatment with an initial concentration of 2 mg/container resulted in a decrease of 1.99 ± 0.00 mg/container, were a concentration of 0.12 ± 0.02 mg/container was measured in the plant tissue. The other mixture treatment with an initial concentration of 0.8 mg/container VEN, a decrease of 0.80 ± 0.00 mg/container could be quantified, and 0.12 ± 0.02 mg/container could be detected in the plant tissues.

The FLU treatment with a starting concentration of 10 mg/container resulted in a decrease of 9.87 ± 0.00 mg/container, whereas an amount of 0.01 ± 0.00 mg/container was detected in the plant tissues. At a concentration of 4 mg/container, a decrease of 3.87 ± 0.00 mg/container was measured, and an amount of 0.01 ± 0.00 mg/container was detected in the plant tissues. Regarding the mixture treatment with an initial concentration of 2 mg/container FLU, a decrease of 1.88 ± 0.00 mg/container was found, of which a quantity of 0.002 ± 0.001 mg/container was detected in the plants. The mixture treatment with an initial concentration of 0.8 mg/container resulted in a decrease of 0.69 ± 0.00 mg/container, while an amount of 0.002 ± 0.001 mg/container was detected in the plant tissues. In the FLU and VEN treatments it is highly probable that the parent compounds were also degraded due to environmental conditions since a significant decrease was determined in the control containers where there were no plants.

### 3.4. Degradation of Xenobiotics by Crude Plant Extracts

To confirm the effectiveness of the degradation of the studied XBs by *T. pannonicum*, a certain concentration of the different XBs was added to the crude extract of the plants cultivated without XBs. According to the results, large differences could be detected concerning the degradation of the different XBs. After the respective incubation time, the antibiotic SMT was degraded significantly. Comparing the SMT concentration using denatured plant extract as control (5.42 mg L^−1^ SMT) and non-denatured plant extract (1.49 mg L^−1^ SMT), a degradation of 72.52% was determined. The concentrations of the mixture of XBs using denatured enzymes were also significantly higher than those where non-denatured enzymes were used. Furthermore, it was found that the SMT concentration in the non-denatured samples was slightly lower in the mixture of XBs than in the pure SMT sample, but this may be due to the interaction between the different compounds and not due to differences in the biodegradation process.

Since the denatured samples were previously incubated at 95 °C for 15 min, resulting in a loss of enzyme activity, it can be assumed that the SMT decrease between 72.4% and 81.3% (Table 5) is due to the enzymatic degradation. It has already been shown that SMT can be readily degraded using the plant extract of *T. pannonicum*, where they reported that up to 85.4% SMT was degraded [18]. In a later experiment, with a starting concentration of 5 mg L^−1^, a degradation of 85.4% was also found [28]. These results fit well with the findings of this study.

The degradation of the xenobiotic APAP showed no significant difference in all treatments. There was no significant difference between the denatured sample and the non-denatured sample nor the samples with pure APAP and the mixture. For all samples, a value close to the starting concentration was determined. It would be reasonable to assume that the plant enzymes were not able to break down APAP. It can be ruled out that the enzymes of the plant extract were prematurely damaged or denatured since all XBs used the same extract and for example, a high degradation rate could be determined for SMT. However, previous experiments have found that enzymatic degradation of APAP by plant enzymes is possible and even undergoes a similar metabolic process as in mammals. In other experiments with cell cultures of *A. rusticana*, where the plants were incubated with APAP, reported that after 6 h of incubation, in addition to the parent compound, APAP-glucoside, APAP-glutathione and the corresponding cysteine conjugate were detected in the root cells. Conjugation with glutathione occurs exclusively on the NAPQI and requires the activation of APAP by the P450 monooxygenase enzyme complex. These P450 enzymes are known to be present in plants where they catalyze several chemical reactions [43]. In future investigations, it would be necessary to carry out further investigations with APAP with extracts of different plant species, because its degradation may be dependent on specific enzymes.

Regarding the CBZ degradation, no difference was found between denatured and non-denatured samples (Table 5). It might be stated that the plant enzymes would not have been able to degrade CBZ. However, previous studies have been able to demonstrate the degradation of CBZ. For example, some CBZ metabolites such as EP-CBZ, DiOH-CBZ, 2-OH-CBZ and 3-OH-CBZ have been detected in cucumber plants [42]. In this latter experiment, the incubation time as well as the xenobiotic concentrations were lower compared to our experiment, yet a degradation of the substance could be determined. This also could be due to the different plant species (e.g., different enzymes and different concentrations) as well as due to the different environmental conditions.

Concerning VEN and FLU, all samples showed a very low concentration (Table 5). However, no significant differences could be detected between the denatured and non-denatured enzymes. The degradation of VEN and FLU by plant enzymes has not yet been clearly confirmed. In other experiment conducted by Rühmland et al. [44], with different constructed wetland systems, including the floating aquatic macrophytes *Iris pseudacorus*, *Scirpus* spp. and *Carex* spp., it was suggested that the degradation depends on the range and amount of compounds as well as on environmental parameters such as redox conditions, temperature, biodegradable dissolved organic carbon concentration, and the composition and activity of the microbial communities. In this same study, a decrease in VEN concentration was reported, but the authors suggested that it was more likely due to photodegradation. In this context, in another previous study in spinach with a starting concentration of 0.1 mg L^−1^ FLU, a decrease in the control was observed within 7 d. Lam et al. [45] also suggested that the high loss of FLU could be explained by photodegradation during the experiment, which is a photolabile substance with a half-life of 55 h. This suggests that the substance was degraded or transformed independently of plant enzymes. In addition to this hypothesis, it was further suggested that the large decrease in FLU concentration in the medium, in addition to the metabolization by plant enzymes, could also be explained by irreversible sorption in plant tissues [46]. Additionally, since in the described studies, as well as in our study, the experiments were not carried out under sterile conditions, a degradation by microorganisms cannot be ruled out. Therefore, the low concentration in VEN and FLU cannot be attributed solely to enzymatic degradation. It would be necessary to clarify if these both compounds can indeed be degraded to this extent by other physical factors.

## 4. Conclusions

The contamination of aquatic environments due to the high use of pharmaceutical compounds has increased. In this sense, different xenobiotics were found in water samples from three different locations in the Tejo estuary, near Lisbon. After screening the different samples, four xenobiotics could be identified including carbamazepine, fluoxetine hydrochloride, venlafaxine hydrochloride and acetaminophen. Furthermore, under controlled conditions, it was determined that the halophyte *Tripolium pannonicum* can uptake these four xenobiotics and sulfamethazine, used as a model antibiotic, but the degradation depends on the xenobiotic itself. The concentration of xenobiotics in the biomass of the plants cultivated under controlled conditions depends on the concentration and the type of xenobiotic. The highest concentration was found in the case of carbamazepine with a concentration of 13.57 µg g^−1^ FM, while venlafaxine and fluoxetine were found in low concentrations in the plant material. According to the mass balance in the culture system and enzymatic test, the degradation of xenobiotics also depends on the kind of xenobiotic and concentration in the culture medium. For example, in the case of Sulfamethazine used as model antibiotic a degradation between 54% and 71% was determined in the hydroponic culture system while in the enzymatic test a degradation between 72.4% and 81.3% was confirmed. Therefore, *T. pannonicum* has a great potential for the phytoremediation of xenobiotics with relatively high efficiency, which could also be used in different saline environments including wetlands for water treatment.

## Figures and Tables

**Table 1:** Retention time and precursors of the xenobiotic standards analyzed by LC-MS.

Standard	Molar Mass [g/mol]	Precursor (ESI+)	Retention Time [min]
Acetaminophen	151.16	152.07	8.34
Azithromycin (dehydrate)	748.99 (785.03)	749.51	31.63
Bezafibrate	361.82	362.11	25.04
Carbamazepine	236.27	237.10	26.34
Citalopram (hydrobromide)	324.39 (405.31)	325.17	28.34
Fluoxetine (hydrochloride)	309.33 (345.79)	310.14	31.91
Gabapentin	171.24	172.13	9.40
Gemfibrozil	250.33	251.2	35.5
Ibuprofen	206.28	n.d.	n.d.
Oxytetracycline (hydrochloride)	460.43 (496.90)	n.d.	n.d.
Propranolol (hydrochloride)	259.34 (295.80)	260.17	27.48
Sulfadoxin	310.33	311.08	8.25
Sulfamethazine	278.33	279.16	12.63
Sulfathiazole	255.32	256.02	8.52
Tetracycline (hydrochloride)	444.44 (480.90)	n.d.	n.d.
Topiramate	339.36	357.13	21.26
Venlafaxine (hydrochloride)	277.40 (313.86)	278.2	27.83

n.d. = not detected.

**Table 2:** Xenobiotic concentration in the water samples from the different sampling sites in Tejo estuary, Lisbon.

Xenobiotic	Xenobiotic Concentration [ng L^−1^]		
	Seixal	Rosario	Alcochete
Acetaminophen	81.0	6.04	<LOD
Azithromycin dhydrate	<LOD	<LOD	<LOD
Bezafibrate	<LOD	<LOD	<LOD
Carbamazepine	5.86	<LOD	<LOD
Citalopram hydrobromide	<LOD	<LOD	<LOD
Fluoxetine hydrochloride	88.46	68.16	68.28
Gabapentin	<LOD	<LOD	<LOD
Propranolol hydrochloride	<LOD	<LOD	<LOD
Sulfadoxin	<LOD	<LOD	<LOD
Sulfamethazin	<LOD	<LOD	<LOD
Sulfathiazole	<LOD	<LOD	<LOD
Topiramate	<LOD	<LOD	<LOD
Venlafaxine hydrochloride	25.8	13.68	<LOD

<LOD = below limit of detection.

**Table 3:** Xenobiotic concentration in shoots in *Tripolium pannonicum*. The values 5 and 2 in the table header represent the singly xenobiotic concentration and the values 1 and 0.4 represent each xenobiotic concentration in a mixture of the five xenobiotics in mg L^−1^. Values show the mean (in µg g^−1^ FM) ± standard error of three biological replicates per treatment.

Shoots	Initial Concentration (mg L^−1^)			
Xenobiotic	5 (Singly)	2 (Singly)	1 (Mixture)	0.4 (Mixture)
Sulfamethazine	1.58 ± 0.30 C	1.09 ± 0.15 C	0.47 ± 0.05 C	0.53 ± 0.07 C
Acetaminophen	1.19 ± 0.44 C	0.33 ± 0.01 C	1.42 ± 0.26 C	1.93 ± 0.31 C
Carbamazepine	13.57 ± 0.71 A	6.98 ± 1.85 B	4.99 ± 0.18 B	2.23 ± 0.83 C
Venlafaxine	0.59 ± 0.22 C	0.47 ± 0.08 C	0.34 ± 0.07 C	0.09 ± 0.03 C
Fluoxetine	0.20 ± 0.02 C	0.13 ± 0.01 C	0.03 ± 0.00 C	0.02 ± 0.00 C

Mean values with the same letter are not significantly different (*p* > 0.05).

**Table 4:** Xenobiotic concentration in roots in *Tripolium pannonicum.* The values 5 and 2 in the table header represent the singly xenobiotic concentration and the values 1 and 0.4 represent each xenobiotic concentration in a mixture of the five xenobiotics in milligram per litre. Values show the mean (in µg g^−1^ FM) ± standard error of three biological replicates per treatment.

Roots	Initial Concentration (mg L^−1^)			
Xenobiotic	5 (Singly)	2 (Singly)	1 (Mixture)	0.4 (Mixture)
Sulfamethazine	68.96 ± 3.28 A	30.20 ± 1.56 B	12.66 ± 1.65 C	6.41 ± 0.64 DE
Acetaminophen	9.74 ± 1.74 CD	7.37 ± 0.41 CDE	6.42 ± 0.69 DE	5.40 ± 0.16 DEF
Carbamazepine	2.14 ± 0.35 EFG	0.49 ± 0.11 FG	0.40 ± 0.09 FG	0.17 ± 0.01 FG
Venlafaxine	0.28 ± 0.21 FG	0.13 ± 0.03 FG	0.05 ± 0.03 FG	0.04 ± 0.01 FG
Fluoxetine	0.23 ± 0.13 FG	0.06 ± 0.00 FG	0.00 ± 0.00 G	0.01 ± 0.00 FG

Mean values with the same letter are not significantly different (*p* > 0.05).

**Table 5:** Xenobiotic degradation (mg L^−1^) by crude plant extract. Initial concentration of 5 mg L^−1^. Values show the mean (in mg L^−1^) ± standard error of three replicates per treatment.

Xenobiotic	Single Treatment		Mixture of XBs	
	Not Denatured	Denatured	Not Denatured	Denatured
*Sulfamethazine*	1.4969 ± 0.12 c	5.4227 ± 0.18 a	0.7748 ± 0.14 d	4.1400 ± 0.08 b
*Acetaminophen*	3.8860 ± 0.67 a	4.2600 ± 0.76 a	5.0660 ± 0.34 a	4.4993 ± 0.70 a
*Carbamazepine*	7.0553 ± 0.07 a	7.0927 ± 0.03 a	6.7460 ± 0.11 a	6.7933 ± 0.10 a
*Venlafaxine HCl*	0.0397 ± 9.4 × 10^−5^ a	0.0341 ± 9.7 × 10^−4^ b	0.0368 ± 1.2 × 10^−3^ ab	0.0391 ± 1.4 × 10^−3^ a
*Fluoxetine HCl*	0.0205 ± 2.2 × 10^−4^ ab	0.0207 ± 9.6 × 10^−5^ ab	0.0201 ± 8.5 × 10^−4^ b	0.0225 ± 3.4 × 10^−4^ a

Mean values with the same letter are not significantly different (*p* > 0.05).

## Data Availability

Data is contained within the article and supplementary material.

## Supplementary Materials

The following are available online at https://www.mdpi.com/1660-4601/18/3/943/s1, Table S1: Detection and quantification limits of the xenobiotics.
