# Supplementary material for: Screening of Emerging Pollutants (EPs) in Estuarine Water and Phytoremediation Capacity of Tripolium pannonicum under Controlled Conditions
Source: Int J Environ Res Public Health. 2021 Jan 22;18(3):943. doi: 10.3390/ijerph18030943 (PMC7908520; doi:10.3390/ijerph18030943)
Supplement: Supplementary file 1 [file ijerph-18-00943-s001.pdf]

Supplementary Material

Table S1. Detection and quantification limits of the xenobiotics.

Standard	Detection Limit [ng mL^{-1}]	Quantification Limit [ng mL^{-1}]
Acetaminophen	0.37	1.61
Azithromycin dihydrate	5.25	20.9
Bezafibrate	3.5	14.33
Carbamazepine	3.02	11.75
Citalopram hydrobromide	6.51	25.52
Fluoxetine hydrochloride	4	16.28
Gabapentin	4.41	16.88
Gemfibrozil	n.d.	n.d.
Ibuprofen	n.d.	n.d.
Oxytetracycline hydrochloride	n.d.	n.d.
Propranolol hydrochloride	3.04	12.56
Sulfadoxin	3.68	14.18
Sulfamethazin	6.59	25.79
Sulfathiazole	3.74	15.27
Tetracycline hydrochloride	n.d.	n.d.
Topiramate	2.81	11.67
Venlafaxine hydrochloride	6.23	24.49

n.d. Not detected.
